# Alterations in Cardiac Function Following Endurance Exercise Are Not Duration Dependent

**DOI:** 10.3389/fphys.2020.581797

**Published:** 2020-09-18

**Authors:** Alexandra M. Coates, Trevor J. King, Katharine D. Currie, Joshua C. Tremblay, Heather L. Petrick, Joshua T. Slysz, Christopher Pignanelli, Jordan A. Berard, Philip J. Millar, Jamie F. Burr

**Affiliations:** ^1^The Human Performance and Health Research Laboratory, Department of Human Health and Nutritional Sciences, University of Guelph, Guelph, ON, Canada; ^2^Exercise and Cardiovascular Health Outcomes Laboratory, Department of Kinesiology, Michigan State University, East Lansing, MI, United States; ^3^Cardiovascular Stress Response Laboratory, Queen’s University, Kingston, ON, Canada; ^4^Cardiovascular Physiology Laboratory, Department of Human Health & Nutritional Sciences, University of Guelph, Guelph, ON, Canada

**Keywords:** echocardiography, prolonged exercise, ultramarathon, trail running, intensity, preload maintenance

## Abstract

Cardiac function has been shown to transiently decrease following prolonged exercise, with greater durations related to increased impairment. However, the prospective assessment of exercise duration on cardiac performance is rare, and the influence of relative exercise intensity is typically not assessed in relation to these changes. The aim of this study was to determine whether progressively longer running distances over the same course would elicit greater cardiac impairment. The present investigation examined cardiac alterations in 49 athletes, following trail-running races of 25, 50, 80, and 160 km, performed on the same course on the same day. Echocardiography, including conventional and speckle tracking imaging, was performed with legs-raised to 60° to mitigate alterations in preload both pre- and post-race. Race-intensities were monitored via heart rate (HR). Following the races, mean arterial pressure (Δ−11 ± 7 mmHg, *P* < 0.0001), and HR (Δ19 ± 14 bpm, *P* < 0.0001) were altered independent of race distance. Both left and right ventricular (LV and RV) diastolic function were reduced (ΔLV E/A −0.54 ± 0.49, *P* < 0.0001; ΔRV A’ + 0.02 ± 0.04 m/s, *P* = 0.01) and RV systolic function decreased (ΔTAPSE −0.25 ± 0.9 cm, *P* = 0.01), independent of race distance. Cardiac impairment was not apparent using speckle tracking analysis with cubic spline interpolation. While race duration was unrelated to cardiac alterations, increased racing HR was related to greater RV base dilation (*r* = −0.37, *P* = 0.03). Increased time spent at higher exercise intensities was related to reduced LV ejection fraction following 25 km (*r* = −0.81, *P* = 0.03), LV systolic strain rate following 50 km (*r* = 0.59, *P* = 0.04), and TAPSE (*r* = −0.81, *P* = 0.03) following 80 km races. Increased running duration did not affect the extent of exercise-induced cardiac fatigue, however, intensity may be a greater driver of cardiac alterations.

## Introduction

Mass participation in prolonged endurance exercise events has increased greatly over the past few decades (Hoffman, 2016). While recreational athletes report participation in events like ultramarathon to improve physical health (Hoffman, 2016), the physiological consequences of participation are incompletely characterized. From a cardiac perspective, there is evidence that prolonged strenuous exercise may transiently alter both left ventricular (LV) (Middleton et al., 2006; Lord et al., 2018) and right ventricular (RV) (Oxborough et al., 2011; Elliott and La Gerche, 2014) function. This alteration is often termed “exercise induced cardiac fatigue,” and event duration and participant cardiorespiratory fitness are thought to play a role in the severity of the associated change (Middleton et al., 2006; La Gerche et al., 2012). Whether or not these functional alterations are a natural and reversable response to prolonged exercise (Scott and Warburton, 2008), are indicative of structural myocardial damage (La Gerche et al., 2012), or are simply a consequence of altered loading conditions post-exercise (Middleton et al., 2006; Lord et al., 2018), is still contested.

Data from meta-analyses and reviews suggest longer duration events elicit greater cardiac alterations, particularly during systole (Middleton et al., 2006; Oxborough et al., 2010a; La Gerche et al., 2017), however, prospective data to specifically interrogate the effects of exercise duration is limited. Only one study to date has directly examined whether greater distance races of a given type of exercise results in greater cardiac fatigue, and it was found that both half (70.3 mile) and full (140.6 mile) long-distance triathlons induced similar diastolic cardiac alterations, with greater reductions to LV ejection fraction and fractional shortening following the longer event (Whyte et al., 2000). Further, the role of exercise intensity on the occurrence of cardiac fatigue is still under-appreciated. Intensity and duration are inherently related, yet an individual can exert a different relative effort for a given event, and there is indication that exercise intensity may drive cardiac fatigue to a greater degree than duration. In a recent meta-analysis, average intensity during exercise ≥45 min was associated with increased cardiac biomarker release and functional alterations, independent of duration (Banks et al., 2010; Donaldson et al., 2019). Further, in a previous meta-analysis it was shown that LV systolic function may require over 10 h of prolonged exercise before alterations become apparent in trained individuals (Middleton et al., 2006), however, it was demonstrated that 1 h of criterium cycle racing, performed at near-maximal intensity (∼88% of maximal heart rate) by well-trained cyclists, was sufficient to elicit alterations in systolic function under controlled-loading conditions (Stewart et al., 2015). Thus, despite nearly 60 years of research, the exercise load required to elicit the typically observed cardiac alterations indicative of exercise induced cardiac fatigue has not been fully determined. This has implications for future research, and whether or not there are clinical consequences of exercise induced cardiac fatigue.

Following prolonged exercise, alterations in post-exercise cardiac loading conditions, including elevated catecholamines and post-exercise hypotension, make it difficult to determine whether cardiac function is truly impaired, or if changes reflect post-exercise alterations to preload, afterload, and heart rate (HR) (Lord et al., 2018). Dehydration during prolonged exercise has been demonstrated to reduce stroke volume (SV) at rest and during exercise, while twist mechanics are maintained or improved (Stöhr et al., 2011b; Watanabe et al., 2020). Rehydration restores most alterations (Stöhr et al., 2011b), suggesting no impairments to intrinsic LV function. Further, the specific racing conditions such as environmental temperature or elevation will alter loading conditions to different degrees. To offset the effects of dehydration and post-exercise vasodilation, methods including the Trendelenburg position (Hassan et al., 2006), saline infusions (Dawson et al., 2007), and raising the legs above the level of the heart (Hart et al., 2007) have all been adopted to promote venous return and maintain preload during echocardiographic imaging. While these methods do not eliminate the confounding effects of altered loading conditions on cardiac function, most previous research has not employed any such techniques, and therefore the predominate theory that longer exercise durations result in greater cardiac fatigue may in fact be reflective of augmented alterations in loading conditions following longer duration races (Lord et al., 2018). Thus, the purpose of this investigation was to characterize alterations in cardiac function following progressively longer running races performed on the same day and using the same race-course, while promoting venous return via passive leg elevation during echocardiographic assessment. As cardiac fatigue appears dependent on time under load, we hypothesized that indices of cardiac fatigue would present only following longer distance events, with those individuals working at the highest relative exercise intensity for each given duration demonstrating the greatest deviations from baseline.

## Materials and Methods

Sixty-two recreational runners competing in the Sulphur Spring trail running races (Ancaster, ON, Canada) at the 25, 50, 80, and 160 km distances volunteered for this study, which was carried out with the approval of the institutional research ethics board and in accordance with the Declaration of Helsinki. This study is one part of a cluster of studies examining the cardiovascular responses and predictors of performance to ultramarathon trail racing (Coates et al., 2020; King et al., 2020). Participants who were healthy, non-smoking, adults between 18 and 60 years (Table 1) visited the laboratory within the month prior to the race for baseline testing (mean 12 ± 7 days prior to race day), wherein they provided written informed consent prior to measures of anthropometrics, a training and racing history questionnaire, supine brachial blood pressure (BP), resting echocardiography with legs raised, brachial venipuncture to assess hematocrit using a microcapillary reader (Damon/IEC Division, MA, United States), and an incremental running test to exhaustion. Brachial venipuncture was performed in a standard phlebotomy chair, and other hematological measures (interleukin-6, c-reactive protein, and total creatine kinase) obtained are detailed in a separate study (King et al., 2020). In preparation for testing, subjects refrained from caffeine and heavy meals for ≥3 h, alcohol and medications for ≥24 h, and intense exercise for ≥48 h. On the race day, participants were weighed immediately before the race in a mobile laboratory stationed near the finish line. Upon race-completion, a second weight measure was taken, followed immediately by hematocrit taken in the same phlebotomy chair. As soon as there was bed space in the mobile laboratory, participants were assessed with supine brachial BP, and echocardiography with legs raised.

**TABLE 1:** Subject characteristics and running history.

	25 km (*n* = 8)	50 km (*n* = 20)	80 km (*n* = 13)	160 km (*n* = 8)
Sex (n)	M(6), F(2)	M(10), F(10)	M(9), F(4)	M(6), F(2)
Age (yr)	42 ± 10	40 ± 10	45 ± 9	45 ± 10
Weight (kg)	73.4 ± 9.4	71.4 ± 15.6	76.1 ± 17.3	71.9 ± 10.9
Height (cm)	177 ± 6	171 ± 9	177 ± 12	172 ± 8
BMI (kg/m^2^)	23.5 ± 2.6	24.2 ± 3.6	24.0 ± 3.3	24.1 ± 2.3
VO_2_max (ml.kg^–1.^min^–1^)	53.4 ± 6.3	49.7 ± 10.6	48.0 ± 3.8	53.8 ± 8.3
Training Years (yr)	6 ± 4	6 ± 4	9 ± 9	12 ± 6
Mean running volume in last year (km/wk)	35.9 ± 22.9	52.7 ± 20.6	55.9 ± 17.0	72.1 ± 18.7*
Mean training (h/wk)	8.9 ± 3.8	10.8 ± 3.8	10.2 ± 2.6	12.6 ± 3.5
Marathons/Ultramarathons completed (n)	7 ± 8	7 ± 5	14 ± 10	20 ± 9_*†_

Data as Mean SD. *P < 0.05 from 25 km group. ^†^P < 0.001 from 50 km group. M, male; F, female; BMI, body mass index.

### Exercise Exposure

**FIGURE 1:**
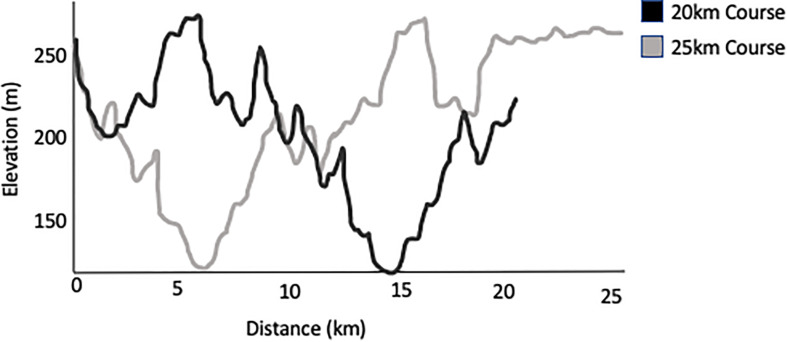
Profile of elevation for a single 20 or 25 km lap of the Sulphur Springs race course. 20 km loops were used for laps of the longer distance races.

The Sulphur Springs course was comprised of a GPS verified 20-km hilly trail circuit that athletes repeated to complete their target distance (e.g., 160 km participants completed eight laps). Each lap consisted of 620 m of elevation, which can be seen in Figure 1. The 25 and 50 km participants completed slightly modified courses to reach their race distances. Running commenced between 6 and 7:30 am with a cut-off at 12 pm the following day (30 h) for the 160 km distance. Participants wore coded HR monitors to track relative exercise intensity.

### Physiological Testing

In the month prior to the race, an incremental running test with indirect calorimetry was performed (HP Cosmos Treadmill, Cosmed Quark metabolic cart) to determine maximal oxygen consumption (VO_2_max), ventilatory thresholds, and associated HR zones. Participants wore a Garmin chest strap to obtain HR (Garmin, Olathe, KS, United States) which was synced via ant + telemetry to the metabolic cart. The athletes also wore their personal HR straps and/or watches that they intended to wear during the race to ensure that the personal HR monitors provided accurate data. Participants performed three, 3 min submaximal stages with a 1% grade to simulate outdoor running (Jones and Doust, 1996), at paces that increased by 0.22 m/s (0.5 mph) per stage from what was estimated to be a sustainable steady pace. Following the three submaximal stages, speed was increased by 0.17 m/s per minute (0.4 mph) or incline was increased by 1% per minute as preferred by the participant, until volitional exhaustion. The maximal test was designed to assess running economy for an additional study (Coates et al., 2020). Ventilatory thresholds were determined as previously defined by Pallarés et al. (2016), and HR was separated into three zones. The light-intensity zone was determined as HR below the first ventilatory threshold, moderate-intensity zone was HR between the first and second ventilatory-threshold, and the high-intensity zone was HR above the second ventilatory-threshold (Esteve-Lanao et al., 2005). If the participant did not have their own HR monitor, or if the data was not consistent with that obtained during the incremental test, participants were provided with a coded Polar A300 watch and H7 chest strap (Polar, Kempele, Finland).

### Echocardiography

Images were collected in the left lateral decubitus position with legs raised at the hip to 60°, with knees bent, both pre- and post-race. This method was chosen as it has been demonstrated that passive leg raise before exercise only causes a minor alteration in filling compared to supine postures, with unchanged end diastolic volume (EDV), left atrial diameter, mean arterial pressure (MAP), and a moderate increase in early transmitral filling velocity (E) (Hart et al., 2007). However, legs raised post-race mitigates alterations in preload and filling pressures following marathon running (Hart et al., 2007) with unchanged MAP, allowing for greater resolution into intrinsic cardiac alterations. Brachial BP was taken at the left arm in triplicate using an automated oscillometric device (BpTru, Vancouver, BC, Canada) while the subject rested supine prior to passive leg raise. Two-dimensional, M-mode, pulsed-waved Doppler, tissue Doppler imaging, and speckle-tracking echocardiography were performed by two experienced ultrasound technicians using a dedicated ultrasound (Vivid Q, GE healthcare, Horten, Norway) with a M4S Matrix Sector Array Probe (2–5 MHz). The primary sonographer performed all baseline testing and post-race testing, with support by the second sonographer during the 30 h of post-race testing. Images were analyzed offline (GE EchoPAC) by the primary sonographer according to the recommendations of the American and Japanese Societies for Echocardiography (Quiñones et al., 2002; Lang et al., 2005; Mor-Avi et al., 2011). All indices were determined from the average of a minimum of three cardiac cycles from the parasternal and apical windows.

Left ventricular end-diastolic volume (LV EDV) and end-systolic volume (LV ESV) were assessed via the 4-chamber modified Simpson’s formula, which was used to calculate stroke volume (SV) as EDV-ESV, cardiac output (Q) as HR × SV, and ejection fraction (EF) as EF = (EDV−ESV)/EDV. Early and late transmitral filling velocities were assessed using pulsed-wave Doppler at the mitral-valve leaflets. Pulsed-wave tissue Doppler imaging was performed on the right and left lateral free walls at the mitral and tricuspid valve annuli for tissue velocity. Arterial load (E_A_) was estimated using the formula: end-systolic pressure (ESP)/SV, where ESP was determined as 0.9×SBP (Chantler et al., 2008). Left-ventricular end-systolic elastance (E_LV_) was calculated as ESP/ESV (Chantler et al., 2008). Ventricular-vascular coupling (VVC) was then determined as E_A_/E_LV_ (Chantler et al., 2008).

Right ventricular end-diastolic area (RV EDA) and RV end-systolic area (RV ESA) were assessed from the 4-chamber apical view, and RV fractional area change (FAC) was calculated as (EDA- ESA/EDA). RV base diameter at end-diastole was traced at the level of the RV inflow tract. Tricuspid annular plane systolic excursion (TAPSE) was obtained from M-mode of the tricuspid annulus. Tricuspid pulsed-wave Doppler measures were not clear post-race (including RV E/A ratio) and were removed from analysis.

Speckle tracking analysis was performed on parasternal short axis images at the level of the papillary muscles, and in the four-chamber view for LV longitudinal analysis. The traces were analyzed using post-processing software (2D Strain Analysis Tool, Stuttgart, Germany) which performs a cubic spline interpolation, allowing for a normalized temporal display despite alterations in HR. Time to peak strain was assessed as a percentage of the cardiac cycle.

### Statistical Analysis

Statistical analysis was performed using Statistical Package for the Social Sciences software (SPSS version 25; IBM). Data was assessed for normality using the Shapiro–Wilk test and Q-Q plot analysis. Descriptive characteristics, running history information, and exercise duration and intensity were assessed between groups via one-way ANOVA, with Bonferroni corrections for *post hoc* testing. Pre- and post- exercise data was assessed using repeated measures two-way ANOVA with time as a within subject factor, and group as a between subject factor, and Bonferroni corrections for *post hoc* testing. Change scores for bivariate correlational analysis between cardiac alterations and running duration, and time spent in intensity zones, were calculated by subtracting pre-exercise cardiac values from post-exercise values. ΔHR during echocardiography was used as a covariate in an additional analysis for significant findings of traditional echocardiographic measures (non-cubic splined), as increased HR is known to alter both diastolic and systolic parameters (Fredholm et al., 2017), and we were unable to control for HR using exercising-echocardiography. Results are presented as mean ± standard deviation, with alpha set *a priori* at *P* < 0.05. We report a same day within-subject intra-class correlation of 0.95 for LV EDV, and 0.89 for RV end-diastolic base diameter for the primary sonographer.

## Results

Of the 62 subjects tested at baseline, 49 (79%) completed the totality of their respective race distances and were included in the analysis. Post-exercise testing occurred an average of 75 ± 29 min after the race. Temperature varied throughout the day, peaking at highs of 28°C and 55% humidity across the 2 days of continuous racing, with an average of 16°C with 77% humidity overnight. Descriptive participant characteristics and running history information are presented in Table 1.

### Running Duration and Intensity

**FIGURE 2:**
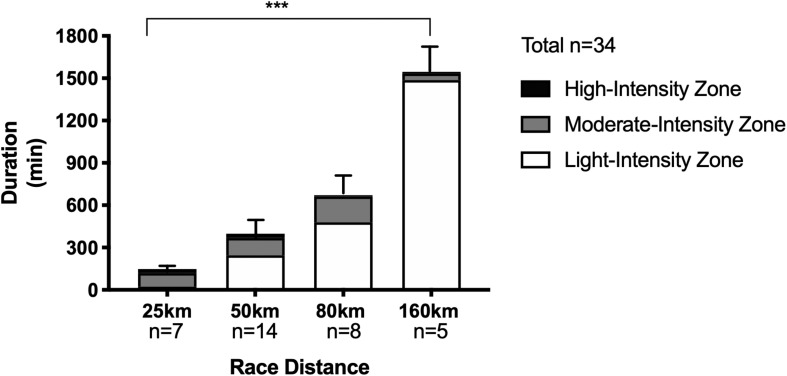
Duration and time spent in individual heart rate intensity zones for trail running races of increasing distances. Data as Mean ± SD for overall duration. ***P < 0.0001 between groups for overall duration.

Mean finishing times for the 25, 50, 80, and 160 km distances were 2.5 ± 0.4, 6.3 ± 1.9, 11.6 ± 1.8, and 25.2 ± 3.6 h, respectively (*P* < 0.0001; see Figure 2). Average exercise intensity (calculated using HR recordings of participants for whom full data was available due to some watches losing power, *n* = 34 obtained) was lower with increasing distance: 164 ± 6 bpm (25 km), 150 ± 12 bpm (50 km), 138 ± 14 bpm (80 km), and 119 ± 23 bpm (160 km) (*P* < 0.0001), as was percentage of the exercise at each given intensity (see Figure 2).

### Hydration Status, Blood Pressure, and Heart Rate

**FIGURE 3:**
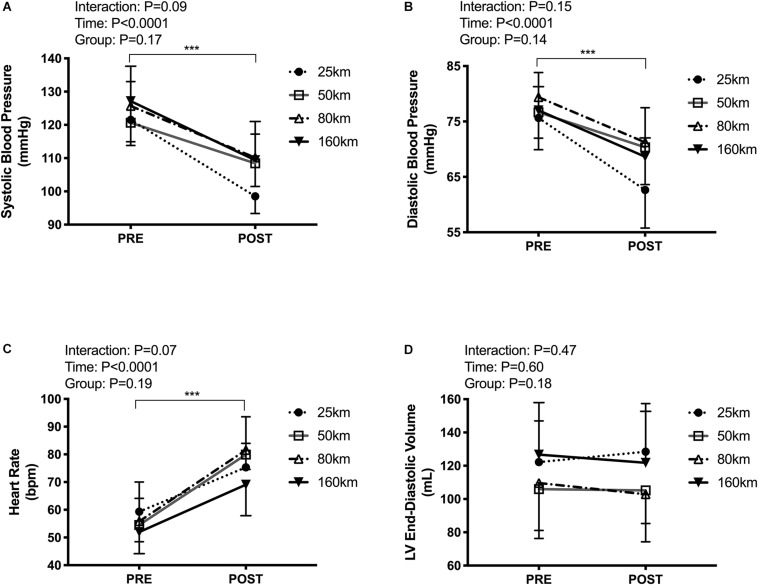
Cardiac loading conditions pre-post trail running races of increasing distances including resting systolic blood pressure **(A)**, diastolic blood pressure **(B)**, heart rate **(C)**, and left ventricular end-diastolic volume **(D)**. Measures were taken in the left lateral decubitus position with legs raised to 60°. ***P < 0.0001, main effect of time.

Participants presented with body mass losses from pre to post-race, with no difference in the magnitude change between groups (Δ−1.6 ± 1.0 kg, *P* < 0.001), and unaltered hematocrit (Δ−0.1 ± 3.3%, *P* = 0.3). Systolic and diastolic BP decreased (systolic:123 ± 11 to 107 ± 9 mmHg, *P* < 0.0001; diastolic:77 ± 7 to 69 ± 7 mmHg, *P* < 0.0001), with no between-group differences. Post-exercise HR was elevated in all groups from 55 ± 9 to 78 ± 12 bpm (*P* < 0.0001), with no between-group differences (see Figure 3).

### Echocardiography

Cardiac function from pre- to post-exercise was not differentially affected when comparing across race distances (no group × time interactions). Irrespective of distance, classic markers of LV diastolic function revealed alterations, including decreased E/A ratio (1.9 ± 0.6 to 1.4 ± 0.5, *P* < 0.001), accelerated late diastolic tissue velocity A’ (0.08 ± 0.02 to 0.10 ± 0.02 m/s, *P* < 0.001), and reductions in LV filling pressure E/e’ (6.3 ± 1.9 to 5.6 ± 1.6, *P* = 0.03), despite unchanged preload under testing conditions (LV EDV 113.4 ± 29.1 to 111.7 ± 28.8 mL, *P* = 0.54). Only E/e’ was no longer significant when ΔHR was used as a covariate. LV systolic function was altered post-exercise such that Q was elevated (3.6 ± 1.1 to 4.7 ± 1.6 L/min, *P* < 0.0001) driven by increased HR (55 ± 9 to 78 ± 12 beats/min, *P* < 0.0001) with unchanged SV (66.8 ± 16.6 to 66.6 ± 18.0 mL, *P* = 0.99). Systolic tissue velocity (S’) was elevated post-exercise (0.09 ± 0.02 to 0.11 ± 0.03 m/s, *P* = 0.002), however, this relationship was no longer significant when adjusting for ΔHR as a covariate (*P* = 0.3). LVEF was unchanged in all race distances (60 ± 8 to 60 ± 8%, *P* = 0.6), while the LV contractility index of SBP/ESV was reduced (3.0 ± 1.1 to 2.7 ± 0.9 mmHg/mL, *P* = 0.04), driven by a decreased pressure (see Table 2). As expected, any change to LVEF was associated with concomitant individual alterations in LV EDV post-race (*r* = 0.53, *P* < 0.0001). Net arterial load (E_A_) was reduced following all race distance (1.8 to 1.5 mmHg/mL, *P* = 0.001), as was ventricular elastance (E_LV_) (2.7 to 2.4 mmHg/mL, *P* = 0.04), resulting in no change to VVC (0.71 to 0.70, *P* = 0.8).

**TABLE 2:** Left ventricular standard, pulsed-wave Doppler, and tissue Doppler echocardiography.

	25 km (*n* = 8)		50 km (*n* = 18)		80 km (*n* = 11)		160 km (*n* = 8)		*P*, main effect of time
	Pre	Post	Pre	Post	Pre	Post	Pre	Post	
**Morphological parameters**									
LV end-diastolic volume (mL)	122 ± 25	128 ± 29	106 ± 30	105 ± 31	110 ± 28	103 ± 18	127 ± 31	122 ± 31	0.60
LV end-systolic volume (mL)	47 ± 10	49 ± 14	44 ± 19	43 ± 16	44 ± 17	42 ± 12	56 ± 22	51 ± 25	0.57
**Global diastolic function**									
Peak E Velocity (m/s)	0.79 ± 0.16	0.66 ± 0.14	0.82 ± 0.13	0.70 ± 0.16	0.74 ± 0.13	0.67 ± 0.11	0.78 ± 0.14	0.76 ± 0.10	0.0001
Peak A Velocity (m/s)	0.42 ± 0.05	0.56 ± 0.10	0.45 ± 0.11	0.54 ± 0.13	0.46 ± 0.15	0.56 ± 0.11	0.42 ± 0.10	0.47 ± 0.09	0.0001
Peak E/A ratio	1.9 ± 0.6	1.2 ± 0.4	2.1 ± 0.7	1.5 ± 0.6	1.7 ± 0.4	1.2 ± 0.3	2.0 ± 0.6	1.7 ± 0.5	0.0001
Deceleration time (ms)	188 ± 18	201 ± 52	179 ± 40	187 ± 42	213 ± 34	179 ± 27	193 ± 45	196 ± 39	0.78
IVRT (ms)	72 ± 14	64 ± 14	74 ± 18	77 ± 21	73 ± 23	74 ± 26	72 ± 20	77 ± 17	0.95
**Global systolic function**									
Ejection fraction (%)	61 ± 8	62 ± 7	60 ± 9	60 ± 8	59 ± 7	60 ± 8	57 ± 9	60 ± 11	0.60
Stroke volume (mL)	76 ± 20	79 ± 21	62 ± 16	63 ± 19	64 ± 15	61 ± 13	71 ± 15	71 ± 14	0.99
Cardiac output (L/min)	4.4 ± 1.1	5.9 ± 1.2	3.4 ± 1.1	4.6 ± 1.6	3.3 ± 1.2	4.2 ± 2.0	3.7 ± 0.8	4.9 ± 1.1	0.0001
**Tissue Doppler**									
Peak E’ lateral (m/s)	0.15 ± 0.04	0.15 ± 0.03	0.13 ± 0.04	0.12 ± 0.05	0.13 ± 0.02	0.13 ± 0.03	0.12 ± 0.03	0.12 ± 0.02	0.64
Peak A’ lateral (m/s)	0.09 ± 0.03	0.11 ± 0.03	0.08 ± 0.02	0.10 ± 0.02	0.07 ± 0.02	0.09 ± 0.02	0.09 ± 0.02	0.10 ± 0.02	0.0001
Peak S’ lateral (m/s)	0.10 ± 0.03	0.11 ± 0.03	0.09 ± 0.02	0.10 ± 0.03	0.09 ± 0.01	0.13 ± 0.04	0.09 ± 0.01	0.10 ± 0.02	0.0001
**LV filling Pressures**									
E/e’ ratio	5.5 ± 1.1	4.5 ± 0.9	6.8 ± 2.5	5.8 ± 1.7	5.8 ± 1.4	5.2 ± 1.6	6.4 ± 1.4	6.6 ± 1.4	0.03
**LV afterload**									
SBP/ESV (mmHg/mL)	2.7 ± 0.6	2.1 ± 0.6	3.2 ± 1.3	2.8 ± 0.9	3.3 ± 1.3	2.9 ± 0.9	2.6 ± 1.0	2.6 ± 1.1	0.04
**Ventricular-Vascular Coupling**									
Arterial Elastance (mmHg/mL)	1.6 ± 0.5	1.2 ± 0.3	1.8 ± 0.4	1.7 ± 0.5	1.9 ± 0.6	1.7 ± 0.3	1.7 ± 0.4	1.4 ± 0.3	0.001
Ventricular Elastance (mmHg/mL)	2.4 ± 0.5	1.9 ± 0.5	2.9 ± 1.1	2.5 ± 0.8	2.9 ± 1.1	2.6 ± 0.8	2.4 ± 0.9	2.4 ± 1.0	0.04
Ventricular-Vascular Coupling	0.66 ± 0.20	0.65 ± 0.20	0.71 ± 0.30	0.71 ± 0.25	0.68 ± 0.20	0.71 ± 0.25	0.79 ± 0.28	0.73 ± 0.36	0.80

*Data as Mean ± SD. IVRT, isovolumetric relaxation time. n, lowest amount of data available per race distance.*

In the RV, late diastolic tissue velocity (A’) was accelerated in similar fashion to the LV (0.13 ± 0.03 to 0.15 ± 0.04 m/s, *P* = 0.01), but was no longer significant when ΔHR was used as a covariate. RV systolic function was reduced as TAPSE decreased from 2.9 ± 0.4 to 2.7 ± 0.5 cm (*P* = 0.01), while RV FAC was unchanged (40.4 ± 6 to 38.1 ± 8%, *P* = 0.14; Table 3.).

**TABLE 3:** Right ventricular standard and tissue Doppler echocardiography.

	25 km (*n* = 8)		50 km (*n* = 16)		80 km (*n* = 11)		160 km (*n* = 8)		*P*, main effect of time
	Pre	Post	Pre	Post	Pre	Post	Pre	Post	
**Standard Measures**									
End-diastolic diameter (cm)	4.4 ± 0.7	4.0 ± 0.5	4.2 ± 0.4	4.0 ± 0.4	4.1 ± 0.4	4.0 ± 0.3	4.2 ± 0.5	4.4 ± 0.2	0.08
TAPSE (cm)	3.0 ± 0.4	2.6 ± 0.3	2.9 ± 0.4	2.7 ± 0.5	2.8 ± 0.4	2.6 ± 0.5	3.0 ± 0.6	3.0 ± 0.5	0.01
RV FAC (%)	41.3 ± 4.1	37.3 ± 6.5	41.3 ± 5.3	38.2 ± 7.0	39.5 ± 6.1	40.4 ± 7.3	39.0 ± 8.7	35.3 ± 11.9	0.14
**Tissue Doppler**									
Peak E’ RV (m/s)	0.15 ± 0.03	0.13 ± 0.03	0.14 ± 0.03	0.11 ± 0.07	0.14 ± 0.02	0.12 ± 0.06	0.14 ± 0.02	0.15 ± 0.03	0.08
Peak A’ RV (m/s)	0.13 ± 0.03	0.15 ± 0.03	0.13 ± 0.03	0.15 ± 0.04	0.14 ± 0.03	0.15 ± 0.05	0.14 ± 0.03	0.16 ± 0.04	0.01
Peak S’ RV (m/s)	0.14 ± 0.03	0.15 ± 0.02	0.14 ± 0.02	0.14 ± 0.03	0.14 ± 0.03	0.15 ± 0.02	0.14 ± 0.02	0.15 ± 0.03	0.15

*Data as Mean ± SD. TAPSE, tricuspid annular plane systolic excursion; RVFAC, right ventricular fractional area change. n = lowest amount of data available per race distance.*

Using speckle tracking for cardiac mechanics, there were no post-exercise differences in LV peak strain or time to peak strain. Independent of race duration, some alterations were observed in peak strain rate, including increases to mid-LV radial systolic strain rate, and LV longitudinal systolic strain rate (see Table 4).

**TABLE 4:** Left ventricular peak strain and strain rates.

	25 km (*n* = 8)		50 km (*n* = 19)		80 km (*n* = 13)		160 km (*n* = 8)		*P*, main effect of time
	Pre	Post	Pre	Post	Pre	Post	Pre	Post	
**Peak Strain (%)**									
Longitudinal Strain	−18.4 ± 1.7	−18.2 ± 2.1	−18.8 ± 2.1	−19.0 ± 3.5	−19.0 ± 2.7	−18.7 ± 3.7	−20.4 ± 2.4	−19.0 ± 3.5	0.47
Mid Radial Strain	36.3 ± 11.2	39.8 ± 19.5	31.4 ± 17.0	32.6 ± 16.9	37.5 ± 15.6	30.1 ± 8.6	35.9 ± 13.5	34.0 ± 22.5	0.73
Mid Circumferential Strain	−14.4 ± 2.9	−13.9 ± 4.2	−14.1 ± 4.8	−14.3 ± 3.7	−14.4 ± 3.5	−13.6 ± 5.3	−15.9 ± 3.6	−12.4 ± 5.2	0.10
**Peak Systolic Strain Rates (s^–1^)**									
Longitudinal Strain Rate	−0.96 ± 0.15	−1.00 ± 0.07	−1.01 ± 0.14	−1.11 ± 0.26	−0.97 ± 0.15	−1.15 ± 0.20	−1.05 ± 0.13	−1.09 ± 0.24	0.03
Mid Radial Strain Rate	1.77 ± 0.56	2.68 ± 1.07	1.66 ± 0.67	2.27 ± 0.83	1.85 ± 0.40	2.27 ± 0.86	1.74 ± 0.26	1.91 ± 1.05	0.01
Mid Circumferential Strain Rate	−0.84 ± 0.19	−0.89 ± 0.21	−0.96 ± 0.27	−1.04 ± 0.24	−0.88 ± 0.27	−0.92 ± 0.25	−0.90 ± 0.13	−0.92 ± 0.25	0.09
**Peak Early Diastolic Strain Rates(s^–1^)**									
Longitudinal Strain Rate	1.31 ± 0.25	1.21 ± 0.38	1.32 ± 0.33	1.26 ± 0.42	1.35 ± 0.43	1.33 ± 0.51	1.43 ± 0.38	1.32 ± 0.27	0.36
Mid Radial Strain Rate	−2.31 ± 0.68	−2.01 ± 0.61	−1.84 ± 1.01	−1.90 ± 0.79	−1.87 ± 0.38	−1.89 ± 0.92	−2.57 ± 1.04	1.94 ± 0.60	0.14
Mid Circ Strain Rate	1.22 ± 0.23	0.96 ± 0.22	1.08 ± 0.35	1.08 ± 0.28	1.13 ± 0.33	1.05 ± 0.44	1.09 ± 0.25	1.07 ± 0.53	0.13
**Time to Peak Strain (%)**									
TTP Longitudinal Strain	99.6 ± 5.5	101.9 ± 3.5	99.8 ± 3.2	98.6 ± 3.5	99.2 ± 4.4	100.5 ± 6.2	101.4 ± 5.5	100.8 ± 5.7	0.67
TTP Mid Radial Strain	99.3 ± 10.2	102.8 ± 17.0	112.8 ± 16.8	106.8 ± 17.8	100.2 ± 7.7	98.5 ± 15.6	96.8 ± 14.0	96.8 ± 15.3	0.78
TTP Mid Circumferential Strain	99.5 ± 2.1	100.1 ± 1.7	99.2 ± 1.6	99.2 ± 3.0	101.2 ± 5.4	105.0 ± 11.6	100.7 ± 1.1	101.6 ± 6.6	0.18

*Data as Mean ± SD. TTP, time to peak. n = longitudinal data. Lowest n for radial and circumferential data was (25 km, n = 8; 50 km, n = 13; 80 km, n = 12; 160 km, n = 6).*

Subject characteristics including age, weight, BMI, VO_2_max, training hours, km/wk, years running, and previous number of long-distance races completed, were not predictive of post-exercise cardiac alterations, with the exception of training h/wk being moderately associated with ΔTAPSE (*r* = 0.32, *P* = 0.03), and baseline BMI being inversely related to ΔLVEF (*r* = −0.33, *P* = 0.03). Loss of bodyweight throughout the race and changes to hematocrit were not correlated to cardiac alterations.

### Cardiac Relationships With Exercise Duration and Intensity

**FIGURE 4:**
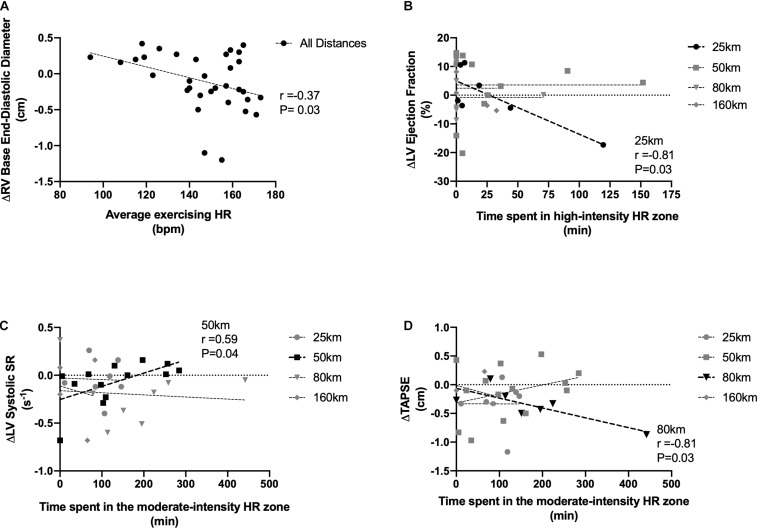
Associations between exercising intensity and RV base end-diastolic diameter **(A)**, LV ejection fraction **(B)**, LV systolic strain rate **(C)**, and RV tricuspid annular plane systolic excursion **(D)**. Exercise intensity expressed as average racing HR **(A)**, time spent in relative high-intensity HR zones **(B)**, and time spent in relative moderate-intensity zones **(C,D)**. Significant relationships are shown in black.

Pooling across all exercise exposures, overall running duration was unrelated to any cardiac value. Using average exercise HR as a marker of intensity, there was an inverse relationship between HR and RV end-diastolic base diameter (*r* = −0.37, *P* = 0.03). In only the 25 km race, LVEF was reduced in those who spent the greatest percentage of exercise time in the high-intensity zone (*r* = −0.81, *P* = 0.03). In the 50 km group, which represented a longer duration and a balance between high/moderate and light intensity exercise, reductions to LV systolic strain rate were related to increased time spent in the moderate intensity zone (*r* = 0.59, *P* = 0.04). In the 80 km event, reductions to TAPSE were related to time spent in the moderate zones (moderate-intensity: *r* = −0.81, *P* = 0.03). There were no relationships to intensity in the 160 km race. Relationships can be seen in Figure 4. Lastly, there also was no relationship between the amount of time from race-finish to post-race imaging and any measure of cardiac function, nor changes with HR or BP.

## Discussion

This investigation sought to characterize the exercise load required to elicit cardiac impairments following prolonged exercise. In recent meta-analyses, it has been found that following exercise over 2 h in duration, there are reductions to systolic LV longitudinal strain, strain rate, and LV EF, as well as diastolic E/A ratio, and E’ (Middleton et al., 2006; Lord et al., 2016; Donaldson et al., 2019), with longer durations demonstrating greater alterations (Middleton et al., 2006). The RV has also been demonstrated to dilate following exercise over 90 min, resulting in RV dysfunction prior to LV alterations (Elliott and La Gerche, 2014). The major finding of the present investigation was that diastolic parameters of the LV, and both diastolic and systolic measures of the RV, were moderately altered following prolonged exercise, however, contrary to our hypotheses, no differences in post-exercise cardiac function occurred as a result of the race distance despite drastically different running durations and intensities. These findings challenge the concept that cardiac fatigue is duration dependent and suggests common loading or measurement conditions may be driving the majority of the observed cardiac alterations, rather than an intrinsic cardiac impairment. However, athletes who worked at higher relative intensities appeared to experience greater functional alterations, suggesting a greater role for intensity than previously explored.

### Left Ventricular Alterations

Previous literature suggests an intrinsic reduction in left ventricular function following prolonged strenuous exercise, with race duration mediating the occurrence and degree of alteration (Middleton et al., 2006; Lord et al., 2018). In a meta-analysis by Middleton et al. (2006) it was demonstrated that only ultra-distance events >10 h elicited reductions in LVEF in trained individuals. In contrast, LV E/A ratio is reduced following endurance exercise as short as 45 min (Donaldson et al., 2019), and E’ is reduced after 2 h of exercise (Lord et al., 2018).

**FIGURE 5:**
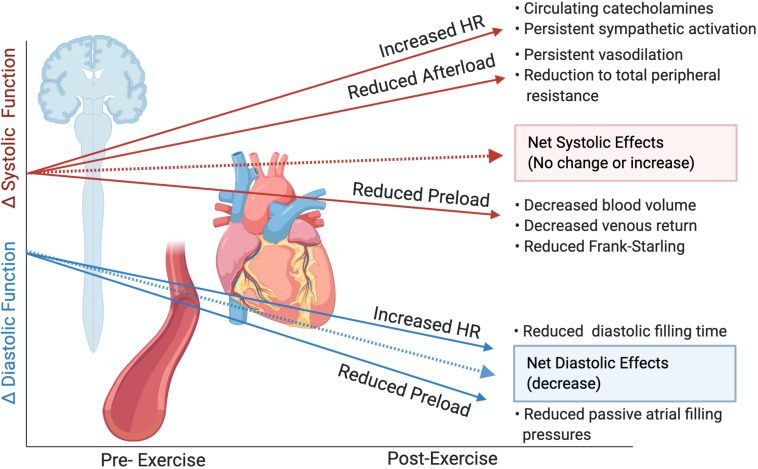
Theoretical alterations to cardiac function following prolonged exercise in the absence of intrinsic myocardial impairments or cardiac fatigue. Slopes are not representative of magnitude differences. Created with Biorender.com.

Left ventricular diastolic function, when assessed via classic measures of E/A ratio and tissue Doppler imaging, was reduced following all exercise durations in the present investigation, as is consistent with previous literature (Middleton et al., 2006; Lord et al., 2018; Donaldson et al., 2019), suggesting that transmitral flow velocities are altered with most forms of aerobic exercise. However, it is understood that elevations in HR reduce the relative time spent in diastole (Oniki et al., 1992), and as such, early-to-late diastolic filling may overlap, making it difficult to separate passive filling from atrial contraction. While others suggest that a lack of correlation between post-exercise changes in HR and changes to E/A ratio indicate an intrinsic impairment to ventricular relaxation (Douglas et al., 1987; Middleton et al., 2006), the persistent and universal elevation of reported HR make it difficult to attribute alterations to the E/A ratio to functional impairments. Further, while we did not assess BP with legs raised (Hart et al., 2007) found no change in MAP following a marathon with legs supine or raised, and we demonstrated greatly reduced BP following all race distances. As such, despite maintenance of preload, reductions to MAP and hence central venous pressure following the races could indicate reduced filling pressures resulting in impaired passive filling, independent of myocardial alterations (Stöhr et al., 2011a) see representative Figure 5). Using speckle tracking with cubic spline interpolation, some of the effects of the shortened cardiac cycle are mitigated (Burns et al., 2010), and we did not see reductions to LV early diastolic strain rate in the present investigation, despite other studies showing reductions following the marathon (Oxborough et al., 2010b) or ultramarathon (Oxborough et al., 2011).

In the present investigation, we did not find evidence of alterations in LV systolic function. While select athletes did experience reductions to LVEF, this reduction was related only to increased time spent in the high-intensity HR zone in the 25 km group. LV systolic alterations have previously been associated with greater exercise intensity and attenuated β-adrenergic sensitivity (Banks et al., 2010), therefore it is possible that even our shortest duration event (25 km and >2 h) represented insufficiently intense exercise to demonstrate between-group LV systolic differences. As LVEF is strongly related to preload, we mitigated post-exercise reductions in preload through passive leg elevation, resulting in no significant changes to LV EDV. Additional systolic parameters considered less load-dependent were also unaltered or were improved following the different exercise exposures. VVC, an index assessing the interaction between the LV and arterial system and considered a measure of net cardiac performance (Chantler et al., 2008), was also unchanged. This occurred despite reductions to E_A_, which is considered a measure of arterial load influenced by afterload (Chantler et al., 2008; Cote et al., 2013), which likely led to a reduced contractile requirement and hence reduced E_LV_. The lack of significant change to systolic parameters suggest intrinsic cardiac inotropy was largely unaltered or was improved by persistent sympathetic drive, and reductions to afterload. As other investigations have also demonstrated no reductions to LVEF with maintenance of preload (Hassan et al., 2006; Dawson et al., 2007; Hart et al., 2007) and LV mechanics have rarely been assessed with preload maintenance, it is likely that post-exercise reductions to preload drive a majority of the purported systolic impairment in the previous cardiac fatigue literature. This is demonstrated in representative Figure 5, whereby in the absence of myocardial alterations, post-race reductions to afterload and increased circulating catecholamines should improve systolic measures, with possible reductions attributable to reduced preload (Stöhr et al., 2011b; Watanabe et al., 2020).

While our current investigation demonstrates mean reductions to only the load and rate dependent measures of LV function, the lack of differences between exercise durations–coupled with the augmented HRs and drastically reduced MAP and hence total peripheral resistance–suggest that there may not be intrinsic LV dysfunction following prolonged exercise. This is in agreement with accumulating evidence that suggests that the LV is largely unaffected by prolonged intense exercise, and that any cardiac fatigue is primarily driven by dilation and dysfunction of the RV (Lord et al., 2016; La Gerche et al., 2017). However, it is also possible that reductions in cardiac performance were occurring during the trail-races that we were unable to capture post-exercise, as two studies to date have demonstrated that imaging performed during exercise reveals greater cardiac impairments (Claessen et al., 2014; Stewart et al., 2015). Stewart et al., demonstrated that when HR and loading conditions were maintained through supine cycling pre and post 1 h of high-intensity cycling, some measures of cardiac fatigue became more apparent during the post-race exercise than at rest (Stewart et al., 2015). This was also seen during cardiac magnetic resonance imaging, whereby cycling during the MRI following a 150 km cycle race demonstrated greater reductions in RV function, without alterations to the LV. Research with further control of hemodynamic and autonomic conditions following prolonged exercise is warranted to delineate these responses.

### Right Ventricular Alterations

It was expected that alterations to RV form and function would occur prior to LV alterations owing to the thinner RV myocardium which is susceptible to dilation, subsequent to increased pulmonary artery pressures during exercise (Elliott and La Gerche, 2014; La Gerche et al., 2017). Further, RV dilation is thought to impinge on LV volumes, resulting in consequent impairments to LV diastolic function (La Gerche et al., 2012). Unfortunately, we were unable to perform a comprehensive assessment of the RV in the present investigation, as the images obtained post-race were often inadequate for analysis. Of the measures obtained, we did not observe changes to be related to exercise exposure, nor were ventricular dilation or reductions of fractional area change evident. However, there were decreases in TAPSE, suggestive of reductions in RV systolic function not present in the LV (Fredholm et al., 2017). Alterations to RV base diameter were also influenced by intensity, which is likely due to greater exposures to elevated pulmonary pressures (La Gerche et al., 2017). It stands to reason that the RV is more susceptible than the LV to exercise-induced fatigue, and while it has been demonstrated that greater exercise durations are related to augmented RV dysfunction (La Gerche et al., 2012), our modest results suggest that that intensity may be a greater driver toward RV impairments compared to duration.

### Limitations

The field-based nature of this study limited the control of some factors, including nutrition and hydration, caffeine use, and non-steroidal anti-inflammatory drug use, however, the benefit of field-based work is the real-world applicability of the findings for athletes. While we attempted to maintain preload with passive leg-raise, due to time constrictions we were unable to also get supine echocardiography as a comparator. Further, stress-echocardiography with supine cycling could have been preferable in order to control HR and autonomic conditions to a greater degree, and future work on the load required to elicit cardiac fatigue will need to control for HR in this fashion. We only included data for which we were confident we had high resolution and accurate image quality and consistency, therefore a comprehensive assessment of the RV and LV short axis views for twist were not included. We did not perform an analysis of sex-differences in the present investigation, as it would only have been possible in our 50 km group due to the low female representation at other distances. Future research should consider higher-intensity events, as it is possible that even our shortest distance event was too long for our recreationally-trained population to see disparate cardiovascular responses. Highly controlled laboratory studies to investigate the effects of intensity versus duration on cardiac alterations are still warranted.

## Conclusion

We sought to determine prospectively whether prolonged strenuous exercise drives reductions in cardiac function through duration and intensity, while controlling for exercise exposure conditions. Unexpectedly, and in contrast with previous literature, we found that all exercise exposures led to similar alterations in cardiac function, suggesting that longer duration events may not induce greater cardiac fatigue. However, we present evidence that intensity may augment potential cardiac alterations. As a reduction to BP and augmented HR were common responses across all participants, and because the observed cardiac alterations are considered to be load and rate dependent, it is likely that these changes to loading conditions drive a majority of the purported cardiac fatigue in the literature to date. Future work using greater intensities of exercise, and controlling for all loading conditions, is needed for a complete understanding of the exercise stimulus required to elicit cardiac fatigue.

## Data Availability Statement

The raw data supporting the conclusion of this article will be made available by the authors, without undue reservation.

## Ethics Statement

The studies involving human participants were reviewed and approved by University of Guelph REB. The patients/participants provided their written informed consent to participate in this study.

## Author Contributions

AC, TK, KC, JT, HP, JS, CP, JB, and JB were involved in the data collection. AC performed the data and statistical analysis, and drafted the manuscript, and revisions were performed by all authors. AC and JB interpreted the data. All authors approved the final version of the manuscript.

## Conflict of Interest

The authors declare that the research was conducted in the absence of any commercial or financial relationships that could be construed as a potential conflict of interest.
